# Diagnostic performance of a doppler radar-based sleep apnoea testing device

**DOI:** 10.1186/s12890-025-03618-9

**Published:** 2025-04-03

**Authors:** Jonathan Röcken, Andrei M. Darie, Leticia Grize, Claire Ellen Dexter, Matthias J. Herrmann, Kathleen Jahn, Werner Strobel, Michael Tamm, Daiana Stolz

**Affiliations:** 1 Clinic of Respiratory Medicine and Pulmonary Cell Research, University Hospital Basel, Basel, Switzerland; 2 Clinic of Respiratory Medicine, University of Freiburg, Freiburg, Germany; 3 Faculty of Medicine, University of Freiburg, Freiburg, Germany

**Keywords:** Home sleep apnoea testing, HSAT, Obstructive sleep apnoea, OSA, Sleepiz, Contactless sleep apnoea testing device

## Abstract

**Background:**

Inpatient polysomnography (PSG) is the gold standard for the diagnosis of obstructive sleep apnoea (OSA), however, both complexity and costs limit the availability of this examination. Home sleep apnoea testing devices are a diagnostic alternative in patients with increased risk of OSA. We evaluated the diagnostic performance of a Doppler radar technology based, contactless sleep apnoea testing device (CSATD) in a cohort of patients with a clinically increased risk of OSA.

**Methods:**

Monocentric prospective study. Sleep monitoring with the CSATD SleepizOne + without pulse oximetry (Sleepiz AG, Switzerland) was performed simultaneously with elective inpatient PSG. PSG was analysed blinded to the CSATD results and according to AASM 2012 criteria by certified sleep physicians. The CSATD data were analysed automatically and independently by a dedicated software.

**Results:**

A total of 102 patients, 60.8% male, with an average age of 55 ± 15 years and body mass index of 30 ± 6 kg/m2 were included in the analysis. The sensitivity and specificity of the CSATD for a PSG apnoea-hypopnoea-index (AHI) of ≥ 5/h were 0.89 (95%CI: 0.83–0.96) and 0.88 (95%CI: 0.73-1.0). The negative and positive predictive values were 0.62 (95%CI: 0.42–0.82) and 0.97 (95%CI: 0.94-1.0). The diagnostic agreement for the diagnosis of OSA (defined as PSG AHI ≥ 5/h) was 89.8% and 100% using a CSATD AHI threshold of ≥ 5/h (*n* = 79/88) and ≥ 15/h (*n* = 61/61). However, the concordance was poor in the classification of OSA severity, with 50% (13/26) concordance for mild, 38% (10/26) for moderate, and 76% (25/33) for severe OSA respectively.

**Conclusion:**

CSATD accurately identifies patients with OSA, particularly using an AHI threshold of ≥ 15/h. However, it performs subpar in disease severity stratification.

**Clinical trial registration:**

This trial was registered on the International Clinical Trials Registry Platform, ISRCTN45778591.

**Supplementary Information:**

The online version contains supplementary material available at 10.1186/s12890-025-03618-9.

## Background

Symptomatic obstructive sleep apnoea (OSA) is common, with a prevalence of 5% in women and up to 14% in men [1]. Left untreated, OSA often impairs quality of life [2] and is associated with an increased cardiovascular risk [3, 4]. If OSA is suspected, sleep evaluation and appropriate diagnostic testing is recommended [5].

Although, in-laboratory overnight polysomnography (PSG) is widely accepted as the reference standard for the diagnosis of OSA, both its complexity and costs might limit access to PSG [5]. The American Academy of Sleep Medicine (AASM) has approved the use of home sleep apnoea testing (HSAT) in selected adult patients, mainly defined as patients with clinically increased risk for moderate to severe OSA and without risk factors for non-obstructive sleep-disordered breathing [5, 6]. HSAT devices offer many advantages, including patient comfort, lower costs, better availability, and prompt analysis [7, 8]. To date, the majority of single or multi-channel devices are worn by the patient and measure parameters such as airflow, (chest) movement, oxygen saturation and/or peripheral arterial tone [5, 9]. Additionally, few contactless sleep apnoea testing devices (CSATD) have been investigated [10]. The main categories of non-contact breathing and sleep monitoring are based on visual [11], audio [12] or radiofrequency technologies [13–16].

SleepizOne + is a new CSATD based on Doppler radar technology [13, 17, 18]. Electromagnetic waves emitted by the device are reflected by the sleeping patients and then analysed, enabling the detection of subtle chest movements. This technique can be used to determine both respiratory rate and heart rate [13, 18].

The objective of this study was to analyse the diagnostic performance of the CSATD (SleepizOne+) in detecting sleep apnoea in a clinical cohort of 102 patients with suspected OSA who underwent in-laboratory PSG.

## Methods

This prospective, monocentric study was performed at the Clinic of Respiratory Medicine and Pulmonary Cell Research at the University Hospital of Basel, Switzerland. The Ethics Committee northwest/central Switzerland approved the study (EKNZ 2018–02086). It was carried out according to the Declaration of Helsinki and Good Clinical Practice guidelines. Patients undergoing in-laboratory PSG at the University Hospital of Basel between May 2020 and January 2021 were approached for study inclusion and only admitted if they gave their informed consent.

The PSGs were analysed by trained sleep physicians blinded to the CSATD results according to the AASM 2012 criteria [19]. The CSATD (SleepizOne+) data were analysed independently and externally by the company (Sleepiz AG, Switzerland), blinded to the PSG results and the patients’ personal data. The CSATD was installed in close proximity to the patient (approximately 40–50 cm) in addition to the standard PSG equipment. It emits an electromagnetic signal at a fixed frequency of 24 GHz which is reflected from the patient’s surface, whereby the duvet does not cause relevant signal interference [13]. The reflected signal is received by the device’s transceiver and processed. Thoracic and abdominal movements, such as breathing, cause small relative distance changes and can be deduced from the reflected signals [13, 18]. The data were analysed by the company’s software after they have been uploaded to a dedicated server. In the present study, the CSATD was not connected to a pulse oximeter. Photographs of the installed device and the reported signal can be found in the supplementary appendix, further information regarding the mechanism of the CSATD can be found in the literature [13, 18].

**Fig. 1:**
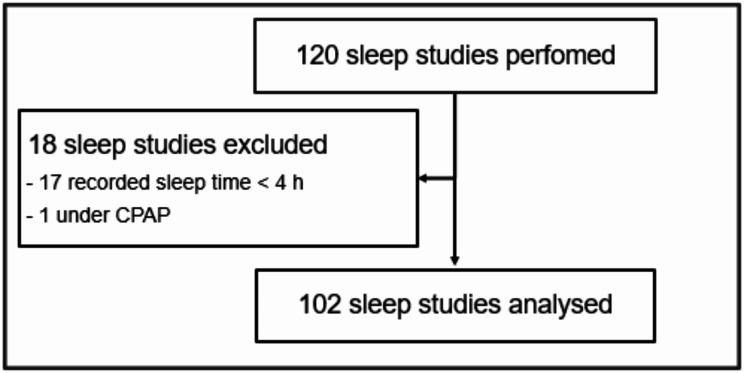
Schematic representation of patient inclusion in the study. Polysomnography and the contactless sleep apnoea testing device (CSATD) were performed simultaneously. CPAP: Continuous positive airway pressure

Proper HSAT diagnostic testing requires at least 4 h of adequate recording [5]. In this study, as shown in Fig. 1, we excluded sleep studies with an estimated total sleep time (TST) of less than 4 h (*n* = 17). Sleep studies under positive airway pressure therapy were also excluded (*n* = 1). Recording was started before the patients went to bed. The total bed time estimate is the period of time during which the device detects that a patient is lying in front of it. The TST is the estimated time spent sleeping within the recording period. All CSATD parameters, including apnoea-hypopnoea-index (AHI), were calculated automatically by the software.

The term sleep apnoea was defined on the basis of an AHI cut-off value of ≥ 5 events/h and classified as mild (5 ≤ AHI < 15/h), moderate (15 ≤ AHI < 30/h) or severe (AHI ≥ 30/h) [5]. The oxygen desaturation cut-off of ≥ 3% was used to determine oxygen desaturation index (ODI) or hypopnoea. It is important to note that the CSATD did not differentiate between central and obstructive apnoea events. Given that only one patient was diagnosed with central sleep apnoea (CSA), the term OSA was used for simplicity and readability. An Epworth Sleepiness Scale (ESS) score of ≥ 11/24 points was used as cut-off for excessive daytime sleepiness [20].

The data, including patient demographics and medical history, were obtained from electronic medical records of the out- and inpatient departments of the University Hospital of Basel. Categorical parameters were summarized as counts and percentages, continuous parameters as means and standard deviation (SD). The Lin’s concordance coefficient and Bland-Altman diagrams were used to examine the agreement between the CSATD and PSG measurements. To evaluate the diagnostic value of the tested method (CSATD) to predict sleep apnoea, receiver operating characteristic curves were drawn. The rate of true positive predictions at different cut-off points were plotted versus the rate of false positive predictions. The area under the curve is reported and the cut-off point with the maximum Youden’s index was chosen to determine the associated sensitivity and specificity of the test. The sleep parameters determined using both instruments were compared using the Wilcoxon’s signed rank test. Generalized linear regression models were performed to determine the effect of body position on the difference between PSG AHI and CSATD AHI. An alpha level of 0.05 was set as the limit of statistical significance. IBM SPSS Statistics for Windows, version 25 (IBM Corp., Armonok N.Y., USA) and SAS version 9.4 (SAS Institute Inc., Cary, NC, USA) were used for the statistical analyses.

## Results

Between May 2020 and January 2021, a total of 120 patients underwent PSG with simultaneous CSATD, of whom 102 were included in the final analysis (Fig. 1).

The patient population was predominantly male (60.8%). The average age was 55.3 ± 15.3 years and the mean body mass index (BMI) was 30.1 ± 6.4 kg/m^2^. Daytime sleepiness, defined by an ESS ≥ 11/24 points, was present in 32.4% (23/71) of the subjects. The most prevalent comorbidities were arterial hypertension (52.0%), chronic kidney disease (CKD 25.5%), depression (17.7%) and diabetes mellitus (16.6%) (Table 1).

**Table 1:** Sociodemographic and clinical characteristics

Characteristics	All (*n* = 102) *n* (%) or mean ± SD,
Age, y	55.3 ± 15.3
Male	62 (60.8)
Body mass index, kg/m^2^	30.1 ± 6.4
Epworth Sleepiness Scale (*n* = 71)	8.1 ± 5.1
*Smoking status* (*n* = 92)	
Current / ex- smoker	48 (52.2)
Never smoker	44 (47.8)
Pack years *	35 ± 30.9
*Comorbidities*	76 (74.5)
Arterial hypertension	53 (52.0)
Asthma	12 (11.8)
Atrial fibrillation	7 (6.9)
Cerebral vascular disease	3 (2.9)
Chronic obstructive pulmonary disease	9 (8.8)
Congestive heart failure	2 (2.0)
Coronary artery disease	14 (13.7)
Depression	18 (17.7)
Diabetes mellitus	17 (16.6)
Renal disease	26 (25.5)
Rheumatological disease	2 (2.0)
Alcoholism	7 (6.9)

* Pack years (defined as the number of cigarettes smoked per day/20 and multiplied by the number of years smoked)

OSA, as diagnosed by PSG, was highly prevalent in this cohort (85/102, 83.3%) (Table 2). The prevalence of mild OSA was 25.5% (26/102) and that of moderate to severe OSA was 57.8% (59/102). The CSATD had an 89.8% agreement with the PSG for the overall diagnosis of OSA (79/88). In all patients with moderate to severe OSA in the CSATD (AHI ≥ 15/h) the diagnosis of sleep apnoea, defined as PSG AHI ≥ 5/h, was confirmed (61/61). Moderate OSA was found in 17.0% (7/41) and severe OSA in 2.4% (1/41) of patients with no or mild OSA in the CSATD.

**Table 2:** Diagnostic performance by OSA severity

	Polysomnography Sleep Apnoea Severity					
CSATD Sleep Apnoea Severity		No	Mild	Moderate	Severe	**Total**
	No	8	3	2	1	14
	Mild	*9*	13	5	0	27
	Moderate	0	6	10	7	23
	Severe	0	4	9	25	38
	**Total**	17	26	26	33	**102**

CSATD: Contactless sleep apnoea testing device. OSA: obstructive sleep apnoea

The area under the receiver operating characteristic curves (ROC AUC) (Suppl. Figure 3) was used to show the diagnostic performance of the CSATD compared to PSG for three different AHI cut-off points. The AUC values for an AHI ≥ 5/h, ≥ 15/h or ≥ 30/h, were 0.92, 0.87 and 0.89, respectively. Table 3 summarizes the performance in terms of sensitivity, specificity, negative and positive predictive values applying these diagnostic thresholds. The sensitivity and specificity of the CSATD for OSA diagnosis (AHI ≥ 5/h) was 89.4% and 88.2% (Table 3).

**Table 3:** Diagnostic performance of the CSATD in the study population

Polysomno-graphy AHI	Sensitivity	Specificity	Negative predictive value	Positive predictive value	AUC
≥ 5/h	0.894 (0.829–0.960)	0.882 (0.729-1.000)	0.625 (0.421–0.819)	0.974 (0.939-1.00)	0.921 (0.869–0.974)
≥ 15/h	0.831 (0.735–0.926)	0.837 (0.727–0.948)	0.783 (0.663–0.902)	0.875 (0.788–0.962)	0.874 (0.802–0.945)
≥ 30/h	0.879 (0.767–0.990)	0.783 (0.685–0.880)	0.931 (0.866–0.996)	0.659 (0.59–0.799)	0.897 (0.825–0.970)

AHI: Apnoea-hypopnoea-index AUC: Area under the curve. CSATD: Contactless sleep apnoea testing device. Numbers in parenthesis are the 95% confidence intervals for the parameter

**Fig. 2:**
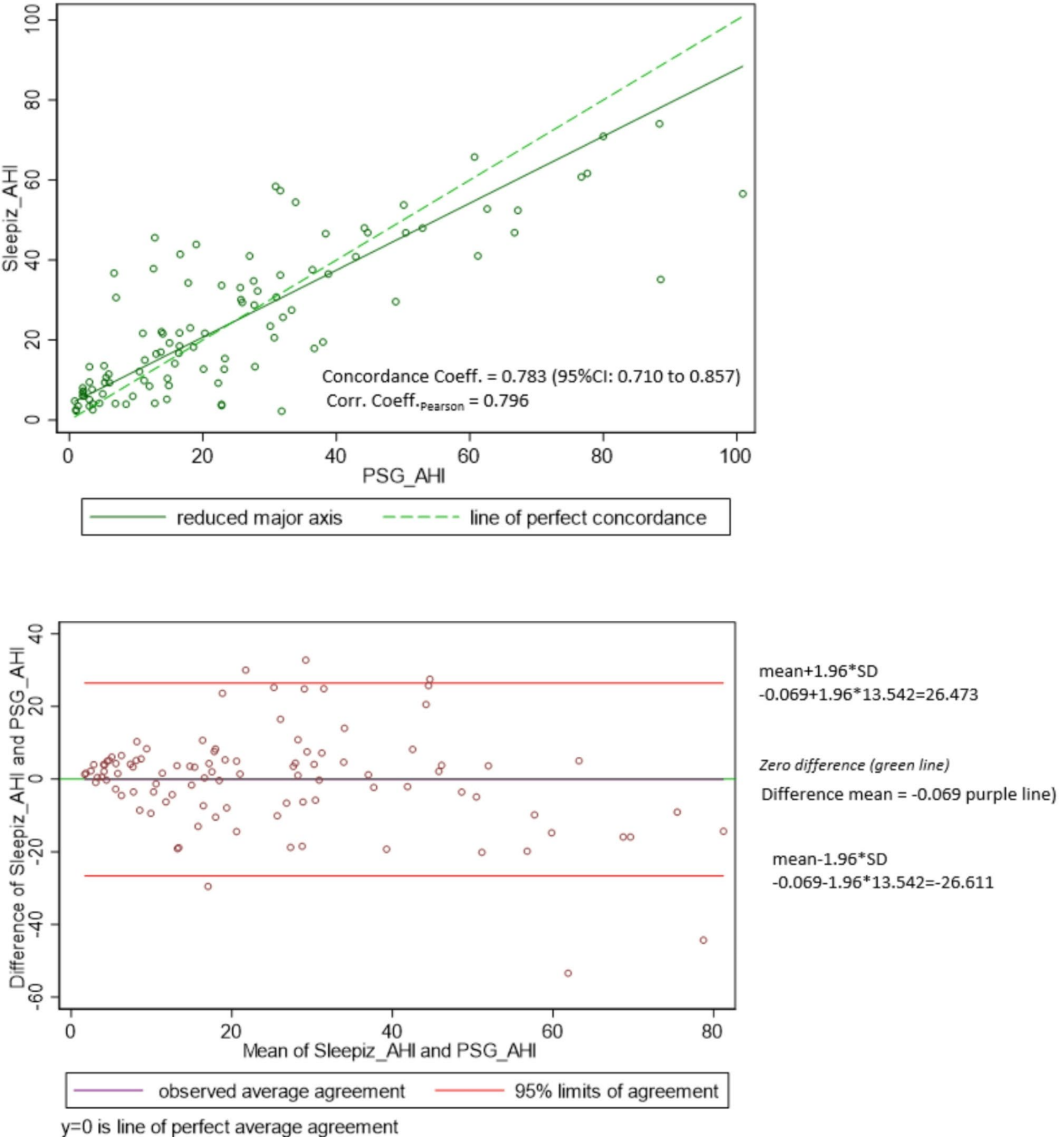
Concordance between AHI measured by the CSATD and polysomnography (**a**) Scatter plot and (**b**) Bland Altman diagram. AHI: Apnoea-hypopnoea-index CSATD: Contactless sleep apnoea testing device. PSG: Polysomnography

The concordance correlation coefficient for AHI measured by PSG and the CSATD was 0.78 indicating a good agreement between both sleep studies (Fig. 2). In the Bland-Altman diagram, we showed that 5.9% (*n* = 6/102) of the data points fall outside the limits of agreement (+ 1.96 SD and − 1.96 SD).

**Fig. 3:**
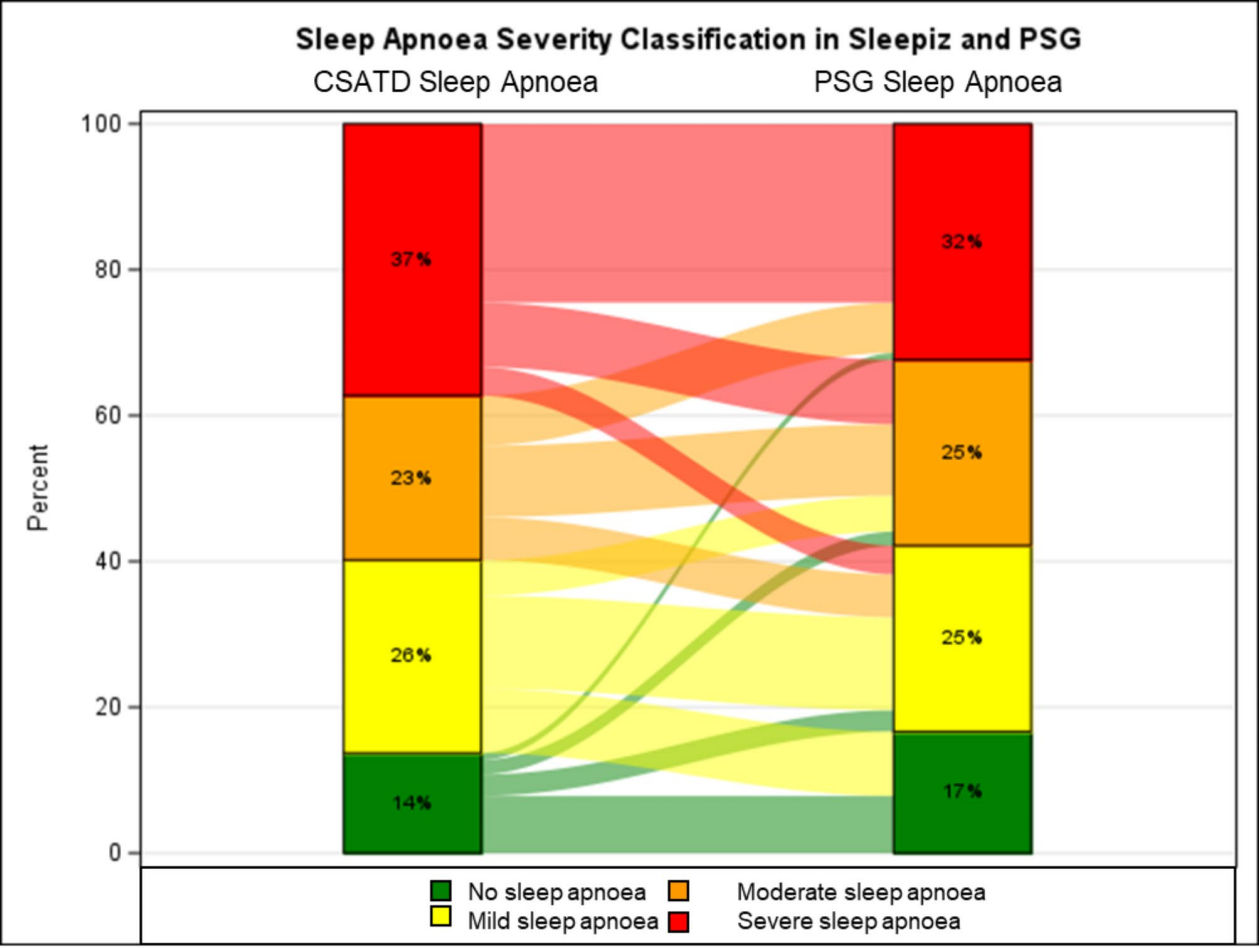
Sankey plot showing sleep apnoea severity classification in both sleep studies (CSATD and polysomnography (PSG)). CSATD: Contactless sleep apnoea testing device

When analysing the concordance with PSG in classifying the severity of OSA, an agreement of 50% (13/26) for mild, 38% (10/26) for moderate, and 76% (25/33) for severe OSA was found (Fig. 3). The ROC AUC analysis showing the performance of the CSATD in classifying OSA severity is provided in Suppl. Figure 4. The AUC values for no sleep apnoea, mild, moderate and severe OSA were 0.92, 0.67, 0.47 and 0.89. Post-hoc analysis of the PSGs of patients with markedly discrepant results, CSATD AHI < 5/h but moderate to severe OSA on PSG, showed that these patients (*n* = 3) had hypopnoea-predominant OSA with an elevated ODI, but an apnoea index of ≤ 6.5/h.

The sleep variables determined with the CSATD and PSG are shown in Suppl. Table 2. A significantly longer mean TST was recorded with the CSATD than with the PSG (368.7 ± 5.8 min vs. 344.9 ± 7.8 min, *p* = 0.006). Mean AHI (24.8 ± 1.8 /h vs. 24.9 ± 2.2 /h) and heart rate (64.9 ± 0.9 bpm vs. 64.5 ± 0.9 bpm, *p* = 0.77) were similar using both diagnostic tools. Suppl. Table 3 provides an overview of the sleep variables that are measured exclusively with PSG. An analysis of these parameters, with a particular focus on sleep-disordered breathing, showed a mean hypopnoea index of 13.0 ± 3.0 /h, an ODI of 24.0 ± 2.1 /h and a mean oxygen saturation of 92.3 ± 0.3%. The majority of patients showed a position-dependent aggravation of AHI. The mean AHI in the supine position was with 36.2 ± 3.1/h markedly higher than the overall mean AHI of 24.9 ± 2.2 /h. In regard to sleep position in percentage of TST, the supine position was predominant with a mean of 53.1 ± 3.0%. The lateral sleeping position was found in an average of 45.7 ± 2.3% of the TST.

Generalized linear regression models were performed to determine the effect of body position on the difference between PSG AHI and CSATD AHI (Suppl. Table 4). Especially the supine position led to an increased AHI difference between the two sleep studies. An increase of 1% in supine position (of TST), increased the difference in AHI by 0.12/h, *p* = 0.0090. It is important to note that the difference also increased with increasing PSG AHI (by 0.33/h per unit increase in PSG AHI, data not shown). When analysing the influence of other parameters on the AHI difference, the hypopnoea index in particular was associated with an increased AHI difference between the two sleep studies. An increase in the hypopnoea index by 1/h increased the AHI difference by 0.75/h (*p* < 0.0001).

## Discussion

The present study analysed the accuracy of the SleepizOne + device, a novel CSATD, for the diagnosis of sleep apnoea in a representative clinical cohort of patients with suspected OSA, in comparison to PSG. We found good agreement in the diagnosis of OSA, especially when using a CSATD AHI cut-off of ≥ 15/h, but discordance in OSA severity stratification or sleep time estimation.

Non-contact sleep monitoring devices are an emerging technology for the assessment of OSA [10, 11, 14–16]. *SleepWise* is based on automatic video analysis for sleep apnoea diagnosis and showed a sensitivity of 100% and a specificity of 83% for an AHI of ≥ 5/h in a trial including 50 patients [11]. Another device, *OrbSense*, monitors sleep related breathing using microwave radar technology [14, 16]. Similarly, *OrbSense* had a 96% sensitivity, but 56% specificity in detecting sleep apnoea compared to PSG. The severity classification was concordant in 69.6% of examinations [16]. *SleepMinder*, a low-power radiofrequency energy device, showed a sensitivity and specificity of 98% and 47%, respectively, using a threshold AHI of ≥ 5/h, whereas the AUC was 0.97 for an AHI ≥ 15/h [15].

Like other non-contact devices, the CSATD analysed in this study showed good diagnostic performance with a sensitivity of 89.4% (95% CI 82.9–96.0), a specificity of 88.2% (95% CI 72.9–100%) and an AUC of 0.92 (95% CI 0.87–0.97) for the diagnosis of sleep apnoea defined by PSG AHI ≥ 5/h. When CSATD AHI is ≥ 15/h, sleep apnoea diagnosis of any severity is likely as it was herein confirmed by PSG in all cases (61/61). Gross-Isselmann et al. also analysed the performance of SleepizOne + compared to PSG in a slightly smaller cohort of patients [18]. They used a binary classification of sleep apnoea with a cut-off AHI of ≥ 15/h to differentiate between moderate to severe sleep apnoea and mild or no sleep apnoea. Using that cut-off, they found a sensitivity of 85.4% and a specificity of 88.1%, which are comparable to our results applying the same cut-off with a sensitivity of 83.1% (CI 73.5–92.6) and specificity of 83.7% (CI 72.7–94.8) [18]. By including data from a pulse oximeter, the device was no longer completely contactless in this study, but showed an increase in sensitivity to 87.8% and specificity to 98.3% [18].

In addition to improved precision, peripheral arterial oxygen saturation measurements provide clinicians with further clinically relevant information, as intermittent oxygen desaturations have been independently associated with cardiovascular disease [21]. In our study we observed three patients without sleep apnoea in the CSATD but moderate to severe sleep apnoea in PSG. These patients showed hypopnoea-dominant sleep apnoea with an increased ODI but an almost normal apnoea index (≤ 6.5/h). Since an increase in the hypopnoea index by 1/h in the PSG increased the AHI difference between PSG and CSATD by 0.75/h, recording the ODI could reduce such discrepant findings or at least alert the physician that sleep-disordered breathing is to be expected.

For uncomplicated patients at increased risk of moderate to severe OSA, the AASM practice guidelines state that OSA can be diagnosed using HSAT devices, which have been shown to have similar sensitivity, specificity and AUC to the CSATD investigated in this study [5]. The potential benefits of HSAT include improved patient comfort, increased availability, and reduced costs [5]. Our data suggest that the diagnosis of OSA can be assumed in patients with a high pre-test probability and moderate to severe findings in the CSATD. However, the low negative predictive value of the CSATD in our population suggests that it may not be sufficient to rule out OSA.

The overall good performance of the device in diagnosing sleep apnoea was tempered by its limited ability to correctly classify the severity of OSA according to the current reference standard, polysomnography (Fig. 3). This was also reflected in a wider limit of agreement in the Bland-Altman diagram (Fig. 2). From a clinical perspective, this represents a considerable limitation of the device. Accurate classification of OSA severity is of clinical relevance as it is important for treatment decisions. In contrast to mild OSA, (moderate to) severe OSA has been shown to be a significant cardiovascular risk factor for patients [22, 23].

Caution is required in patients with suspected or increased risk of CSA, as this device did not differentiate between obstructive and central apnoeas. The effect of CSA on diagnostic accuracy could not be systematically analysed in this study. One patient in this cohort had predominant CSA and was classified as having severe sleep apnoea in both sleep studies.

An important but inherent limitation of our study was the laboratory testing environment, which affected both the installation of the CSATD devices and the patients’ sleep quality and efficiency. It can be assumed that the body position was also influenced by the setting, as the supine position predominated with a mean value of 53.1 ± 3.0% of the TST [24].

Although this is the largest study to date comparing the CSATD with PSG, the generalisability of the results is limited by the single-centre design and the sample size, resulting in a low statistical power of the study (McNemar test, comparison of both sleep studies for OSA diagnosis (Suppl. Figure 5)).

The AASM practice guidelines recommended high pre-test probabilities when a HSAT is performed to diagnose sleep apnoea [5]. A further limitation of our data is that, although the pre-test probability for OSA was clinically assessed, it was not documented in a standardised way. Scores such as the STOP-BANG [25], Berlin questionnaire [26] or NoSAS [27], which are important instruments for assessing the pre-test probability for OSA, could therefore not be provided. The ESS was documented in 69% of the patients. However, the clinical assessment and pre-selection of the patients, some of whom had already undergone HSAT screening tests before being referred for PSG, resulted in a high prevalence of OSA (83.3%) in our clinical population.

## Conclusions

The CSATD performed well compared to PSG in the diagnosis of (obstructive) sleep apnoea in selected patients with clinical suspicion of OSA. Further development, focusing on better sleep severity stratification and differentiation between central and obstructive events, might lead to increased diagnostic options for clinicians in an out-patient setting.

## Electronic supplementary material

Below is the link to the electronic supplementary material.

Supplementary Material 1

## Data Availability

Data is provided within the manuscript or supplementary information files. The datasets used and/or analysed during the current study are available from the corresponding author on reasonable request.

## Abbreviations

AASM: American Academy of Sleep Medicine
AHI: Apnoea-hypopnoea-index
AUC: Area under the curve
BMI: Body mass index
CI: Confidence interval
CPAP: Continuous positive airway pressure
CSA: Central sleep apnoea
CSATD: Contactless sleep apnoea testing device
ESS: Epworth Sleepiness Scale
HSAT: Home sleep apnoea testing
ODI: Oxygen desaturation index
OSA: Obstructive sleep apnoea
PSG: Polysomnography
REM: Rapid eye movement
ROC: Receiver operating characteristic
SD: Standard deviation
TST: Total sleep time
