# Supplementary material for: Diagnostic performance of a doppler radar-based sleep apnoea testing device
Source: BMC Pulm Med. 2025 Apr 3;25:150. doi: 10.1186/s12890-025-03618-9 (PMC11966815; doi:10.1186/s12890-025-03618-9)
Supplement: Supplementary file 1 — Supplementary Material 1 [file 12890_2025_3618_MOESM1_ESM.docx]

**Diagnostic performance of a Doppler radar-based sleep apnoea testing device**

Jonathan Röcken^1^, Andrei M. Darie^1^, Leticia Grize^2^, Claire Ellen Dexter^1^, Matthias J. Herrmann^1^, Kathleen Jahn^1^, Werner Strobel^1^, Michael Tamm^1^, Daiana Stolz^2,3^

**Supplementary appendix**


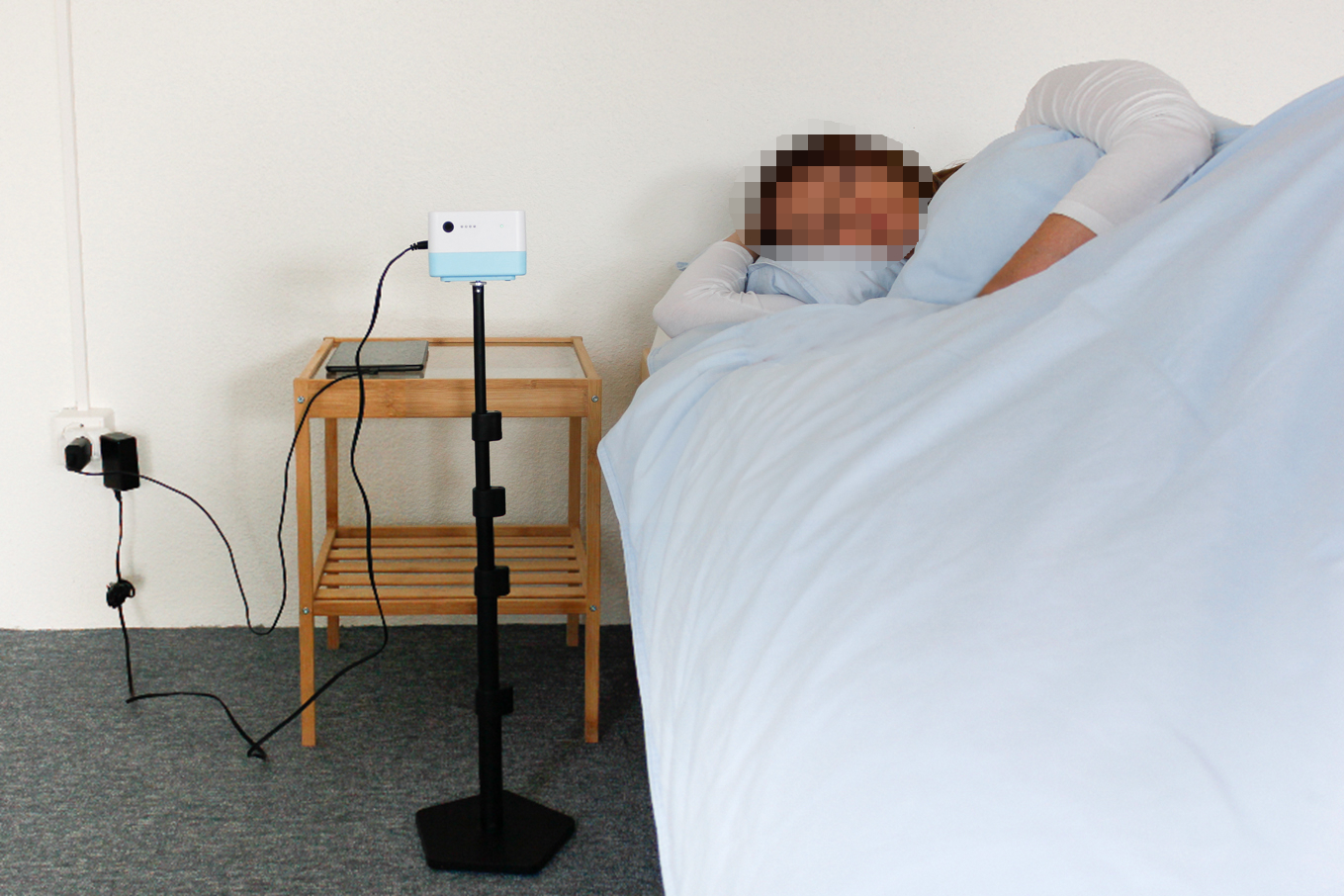


Suppl. Figure 1: The CSATD (installed near the patient) emits an electromagnetic signal at a fixed frequency of 24 GHz which is reflected by the patient's surface. The reflected signal is received by the device’s transceiver and further processed. The photograph was modified and used with the permission of Sleepiz AG, Switzerland.


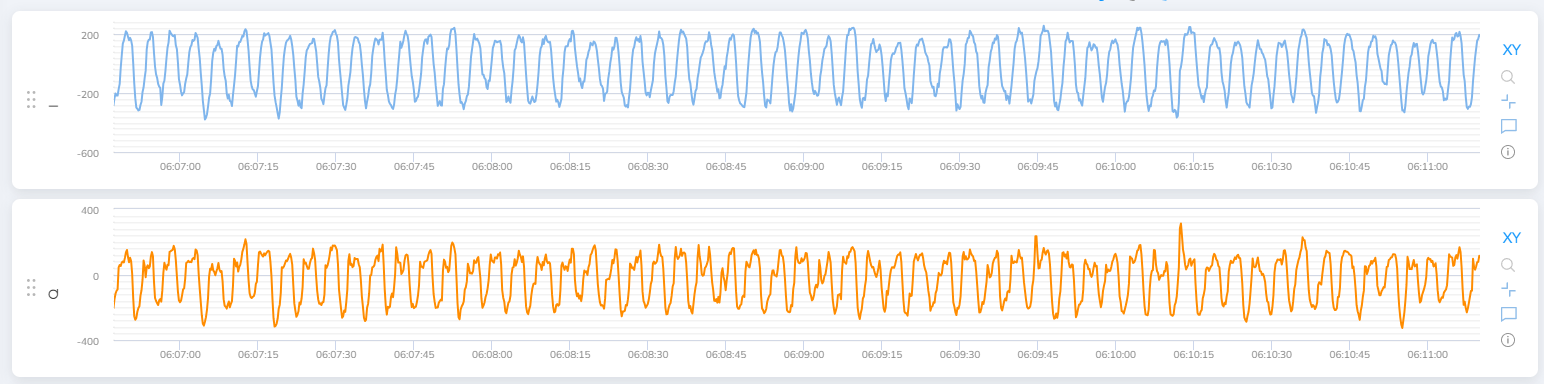


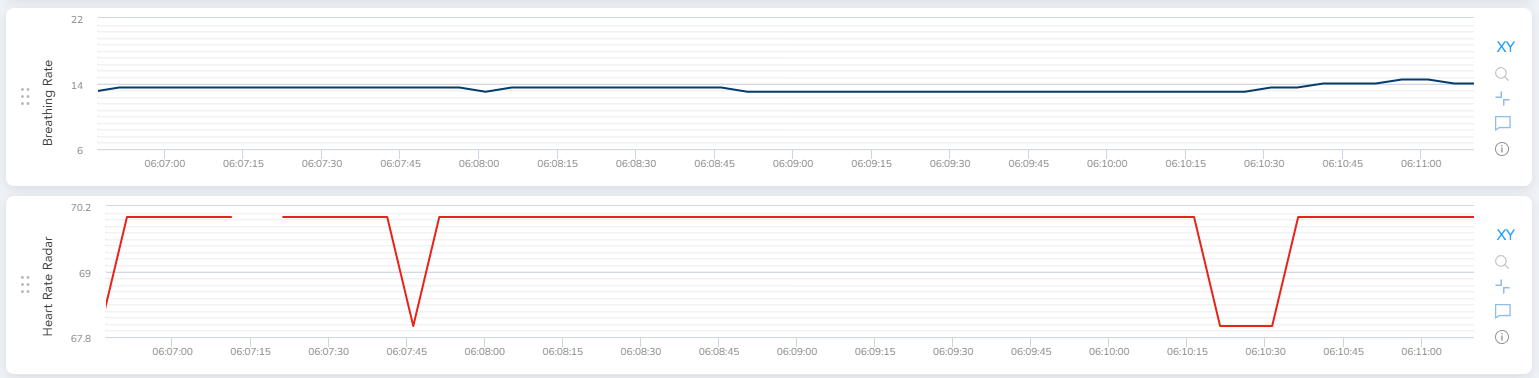


Suppl. Figure 2: The reflected electromagnetic signal is received and processed. The figure shows the raw signal (a, b) and deduced parameters (c, d). a) The in-phase channel (I), b) the quadrature channel (Q) c) the breathing rate and d) the hearth rate. The figure was provided and used with the permission of Sleepiz AG, Switzerland.


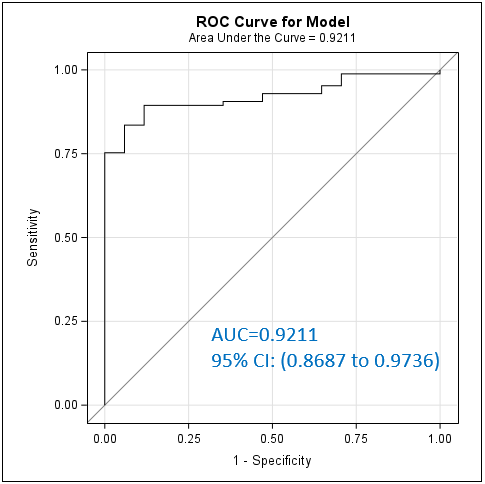

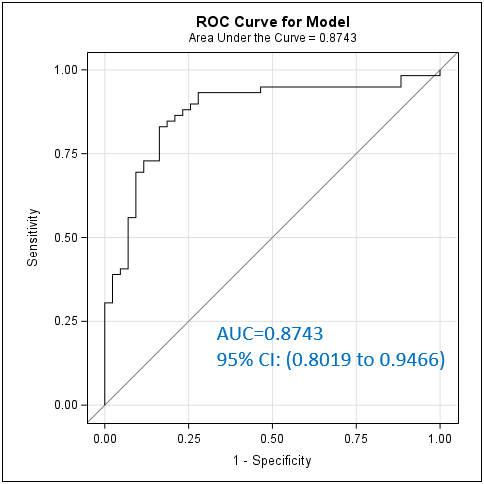


**
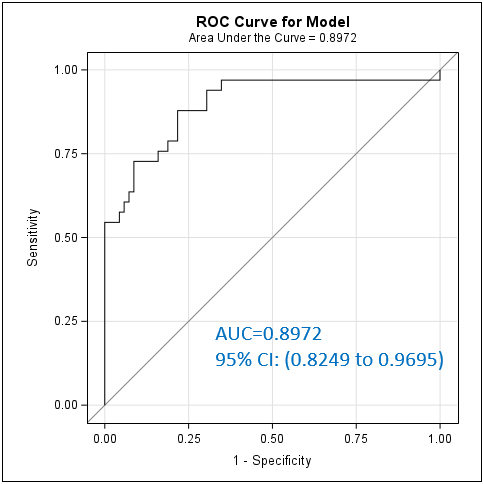
**

Suppl. Figure 3: Receiver-operator characteristics curves for the CSATD estimated AHI versus polysomnography AHI for polysomnography AHI ≥ 5/h, ≥15/h, ≥30/h. AHI: Apnoea-hypopnoea-index AUC: Area under the curve. CSATD: Contactless sleep apnoea testing device.

Suppl. Table 1: Diagnostic performance of the CSATD in the study population.

| **Polysomno-graphy AHI** | **Sensitivity** | **Specificity** | **Negative predictive value** | **Positive predictive value** | **Positive likely-hood ratio** | **Negative likely-hood ratio** | **AUC** |
| --- | --- | --- | --- | --- | --- | --- | --- |
| **≥5/h** | 0.894 (0.829-0.960) | 0.882 (0.729-1.000) | 0.625 (0.421-0.819) | 0.974 (0.939-1.00) | 7.600 | 0.120 | 0.921 (0.869-0.974) |
| **≥15/h** | 0.831 (0.735-0.926) | 0.837 (0.727-0.948) | 0.783 (0.663- 0.902) | 0.875 (0.788-0.962) | 5.102 | 0.202 | 0.874 (0.802-0.945) |
| **≥30/h** | 0.879 (0.767-0.990) | 0.783 (0.685-0.880) | 0.931 (0.866- 0.996) | 0.659 (0.59-0.799) | 4.042 | 0.155 | 0.897 (0.825-0.970) |

AHI: Apnoea-hypopnoea-index AUC: Area under the curve. CSATD: Contactless sleep apnoea testing device. Numbers in parenthesis are the 95% confidence intervals for the parameter.

In contrast to Suppl. Figure 3 and Suppl. Table 1, for Suppl. Figure 4 the ROC curve analysis was performed to analyse the performance of the CSATD to determine the severity of sleep apnoea compared to PSG (no OSA (PSG AHI<5/h), mild OSA (PSG AHI≥5 to <15/h), moderate OSA (PSG AHI≥15 to <30/h) and severe OSA (PSG AHI ≥30/h)).

No OSA: Mild OSA:
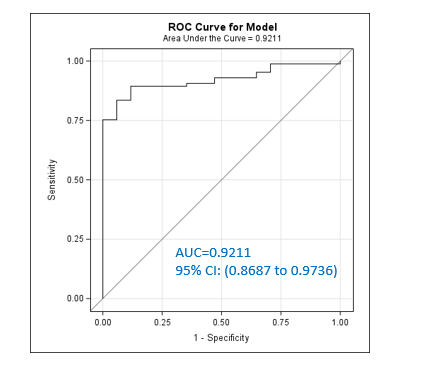

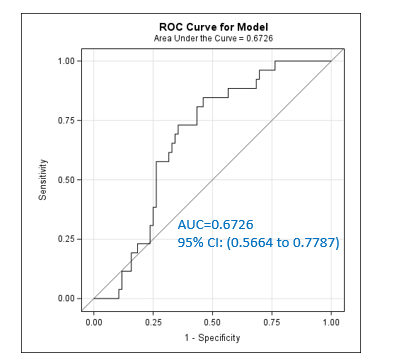


Moderate OSA: Severe OSA:


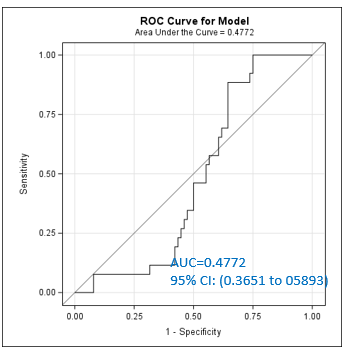

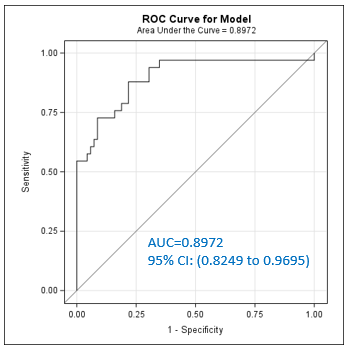


Suppl. Figure 4: Receiver operator characteristic curves to compare the CSATD with the PSG classification of OSA severity. ROC analysis was performed for each degree of severity (no sleep apnoea, mild, moderate and severe OSA) AUC: Area under the curve. CSATD: Contactless sleep apnoea testing device.

The sleep variables determined with the CSATD and PSG are shown in Suppl. Table 2. The total bedtime also differed because the recordings in both sleep studies were not started and ended at the same time. Therefore, the sleep onset latency and sleep efficiency are distorted. The CSATD recorded significantly longer total sleep time. Mean heart rate was similar using both diagnostic tools.

Suppl. Table 2: Comparison of the CSATD and polysomnography sleep parameters.

| **Parameter** | **CSATD; avg±SEM** | **Polysomnography; avg±SEM** | **p value**  Wilcoxon’s signed rank test |
| --- | --- | --- | --- |
| **AHI (/h)** | 24.8±1.8 | 24.9±2.2 |  |
| **Heart rate (beats/min)** | 64.9±0.9 | 64.5±0.9 | p=0.77 |
| **Total Sleep Time (min)** | 368.7±5.8 | 344.9±7.8 | p=0.006 |
| **Sleep Efficiency**  **(Total sleep time/Total bed time (%))** | 67.6±1.0 | 80.2±1.3 | p<0.001 |
| **Sleep Onset Latency (min)** | 69.7±5.5 | 20.0±2.4 | p<0.001 |

AHI: Apnoea-hypopnoea-index CSATD: Contactless sleep apnoea testing device.

Suppl. Table 3: Sleep variables assessed by polysomnography.

| **Parameter** | **Polysomnography; avg±SEM** |
| --- | --- |
| **AHI (/h)** | 24.89 ± 2.21 |
| **Hypopnoea Index (/h)** | 13.05 ± 3.01 |
| **AHI supine position** | 36.22 ± 3.18 |
| **ODI (/h)** | 24.05 ± 2.15 |
| **AHI supine : AHI lateral position** | 4.22 ± 0.45 |
| **Mean oxygen Saturation (%)** | 92.31 ± 0.31 |
| **Snoring Index** | 108 ± 18.60 |
| **Arousal Index (/h)** | 16.52 ± 2.29 |
| **Awake (% Evaluation period)** | 10.24 ± 2.51 |
| **REM Sleep (% TST)** | 15.49 ± 1.06 |
| **Stage N1 Sleep (% TST)** | 15.16 ± 1.18 |
| **Stage N2 Sleep (% TST)** | 53.49 ± 1.26 |
| **Stage N3 Sleep (% TST)** | 18.14 ± 1.19 |
| **Limb movement index (/h)** | 32.14 ± 4.05 |
| **Position in % TST** |  |
| **Back** | 53.17 ± 3.01 |
| **Left** | 22.73 ± 2.39 |
| **Right** | 23.03 ± 2.28 |
| **Prone** | 1.07 ± 0.45 |

AHI: Apnoea-hypopnoea-index, ODI: Oxygen desaturation index, REM: rapid eye movement TST: Total sleep time

Suppl. Table 4: Effect of body position on the difference between PSG AHI and CSATD AHI.

| Outcome | Factor | Estimate | SE | p-value |
| --- | --- | --- | --- | --- |
| PSG AHI – CSATD AHI | Intercept | -6.328 | 2.776 | 0.0249 |
|  | **PSG Supine-Position (% of TST)** | 0.122 | 0.046 | 0.0090 |
| PSG AHI – CSATD AHI | Intercept | 2.513 | 1.906 | 0.1904 |
|  | **PSG Left-Position (% of TST)** | -0.105 | 0.059 | 0.0777 |
| PSG AHI – CSATD AHI | Intercept | 1.190 | 1.996 | 0.5522 |
|  | **PSG Right-Position (% of TST)** | -0.045 | 0.063 | 0.4704 |

Linear model regression. An increase in 1% in PSG Supine-Position, will increase the difference in AHI by 0.122/h, p=0.0090. AHI: Apnoea-hypopnoea-index, CSATD: Contactless sleep apnoea testing device, PSG: Polysomnography, SE: Standard Error, TST: Total sleep time

Suppl. Table 5: Association between various factors and the difference in AHI

| Outcome | Factor | Value | Estimate | SE | p-value |
| --- | --- | --- | --- | --- | --- |
| AHI difference | Hypopnoea Index (PSG) | 1 unit increase | 0.753 | 0.133 | <0.0001 |
| *(PSG AHI – CSATD AHI)* | Age | 1 year increase | -0.104 | 0.093 | 0.2676 |
|  | BMI | 1 unit increase | 0.143 | 0.204 | 0.4853 |
|  | Sex | Male | -2.851 | 2.563 | 0.2692 |
|  |  | Female | 0.000 |  |  |
|  | Limb Movement Index (PSG) | 1 unit increase | -0.053 | 0.031 | 0.0922 |
|  | Atrial fibrillation | Yes | -2.118 | 5.143 | 0.6816 |
|  |  | No | 0.000 |  |  |
|  | Arterial Hypertension | Yes | 1.404 | 2.597 | 0.5903 |
|  |  | No | 0.000 |  |  |
|  | COPD | Yes | -6.062 | 4.163 | 0.1492 |
|  |  | No | 0.000 |  |  |
|  | Congestive heart failure | Yes | -2.495 | 8.373 | 0.7665 |
|  |  | No | 0.000 |  |  |
|  | Intercept | - | -17.228 | 13.422 | 0.2029 |

A generalized linear model regression was performed.

AHI: Apnoea-hypopnoea-index, CSATD: Contactless sleep apnoea testing device, PSG: Polysomnography, SE: Standard Error


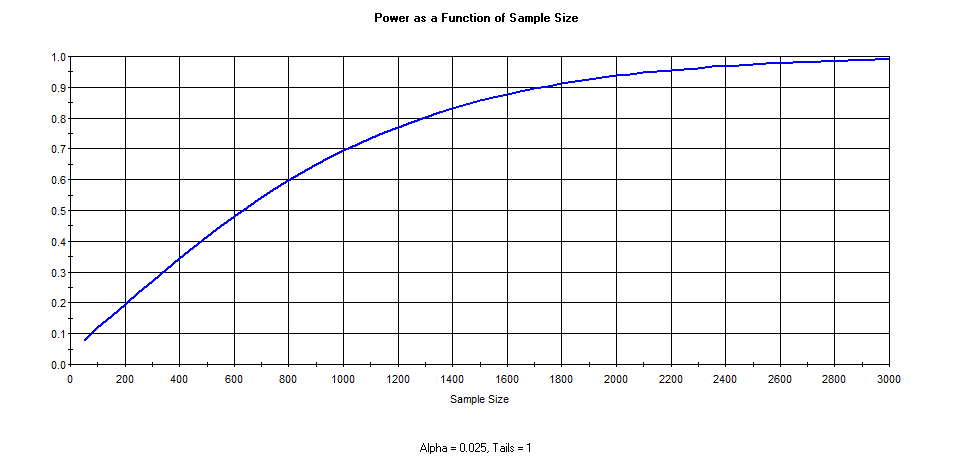


Suppl. Figure 5: Post hoc power calculation for the diagnosis of OSA (yes/no) using the McNemar’s test (one tailed and alpha=0.025) to compare paired proportions. Comparing the CSATD with PSG, the sample size of 102 in our study had a low power of 12.1%. If a power of 80% is required, the sample size would need to be 1,300.
